# A novel retinoic acid drug, EYE-502, inhibits choroidal neovascularization by targeting endothelial cells and pericytes

**DOI:** 10.1038/s41598-023-37619-7

**Published:** 2023-06-27

**Authors:** Yaming Shen, Miao Xu, Ling Ren, Xiumiao Li, Xiaoyan Han, Xin Cao, Jin Yao, Biao Yan

**Affiliations:** 1 The Fourth School of Clinical Medicine, Nanjing Medical University, Nanjing, China; 2 Eye Institute, Eye and ENT Hospital, Shanghai Medical College, Fudan University, Shanghai, China; 3 The Affiliated Eye Hospital, Nanjing Medical University, Nanjing, China; 4 Institute of Clinical Science, Zhongshan Hospital, Fudan University, Shanghai, China

**Keywords:** Medical research, Translational research, Eye diseases

## Abstract

Choroidal neovascularization (CNV) occurs in neovascular age-related macular degeneration (AMD) and often leads to permanent visual impairment. Intravitreal injection of anti-vascular endothelial growth factor (VEGF) agents is the gold standard for the treatment of CNV. However, anti-VEGF treatment did not always cause vision improvement and sometimes had detrimental effects on normal retinal tissues. Herein, we identified a novel retinoic acid drug, EYE-502, which had great therapeutic effects on CNV. Administration of EYE-502 could inhibit VEGF-induced dysfunction of endothelial cells (ECs) and reduce platelet-derived growth factor (PDGF)-induced recruitment of pericytes to ECs in vitro. Administration of EYE-502 could reduce the area of choroidal sprouting and laser-induced CNV, exhibiting similar anti-angiogenic effects as aflibercept. Moreover, administration of EYE-502 could reduce pericyte coverage in the sprouting vessels and choroidal neovascularization. Mechanistically, EYE-502 primarily bound to retinoic acid receptors (RARs) and exerted the anti-angiogenic effects by targeting ECs and pericytes via affecting the activation of Wnt/β-catenin and PDGF/PDGFR/PI3K/Akt signaling. Taken together, this study reports a novel retinoic acid drug, EYE-502, which can exert the anti-angiogenic effects by simultaneous targeting of ECs and pericytes.

## Introduction

Age-related macular degeneration (AMD) is known as a degenerative disease of the central portion of retina that causes vision loss. It has been considered as a major cause of visual impairment. Nowadays, there are nearly 200 million people worldwide suffering from AMD and the number is still increasing^1^. AMD is usually classified into two types: neovascular AMD and non-neovascular AMD. Choroidal neovascularization (CNV) is an important pathological feature of neovascular AMD and the main cause of retinal hemorrhage, exudation, and vision loss^2^. Thus, inhibition of CNV formation is an effective strategy for vision health.

Vascular endothelial growth factor (VEGF)-mediated activation of endothelial cells (ECs) plays a crucial role in the formation of CNV^3^. Intravitreal injection of anti-VEGF agent is one of the main modalities for the treatment of CNV. Some anti-VEGF agents, such as bevacizumab, aflibercept, and ranibizumab, have been proven to effectively inhibit the formation of CNV^4^. However, repetitive injection of anti-VEGF agents can impose great burdens on patients due to monthly doctor visits and potential injury to retinal tissues^5^. Moreover, some patients still experience poor responses to anti-VEGF agents and sustained visual loss following repetitive injections. For example, platelet-derived growth factor (PDGF) is a potential reason for poor responses to anti-VEGF agents. In pathological conditions, due to elevated VEGF levels, ECs proliferate to form microvascular and secrete PDGF-B, which could bind to PDGF receptors (PDGFRs) on the surface of pericytes and recruit pericytes to ECs to form the mature vessels. Previous studies have shown that pericytes play a key role in neovascularization and vascular stabilization^6–8^. The crosstalk between ECs and pericytes contributes to the formation of CNV. Therefore, it is necessary to design a dual-target drug for the treatment of EC and pericyte dysfunction during CNV.

Retinoic acid (RA) is a metabolite derivative of retinol (vitamin A). RA regulates the transcription of target genes with retinoic acid response elements (RAREs) by binding to retinoic acid receptor (RAR) and retinoid X receptor (RXR)^9^. RA plays an important role in cell proliferation, cell differentiation, and cell apoptosis. Previous studies have shown that retinoids can inhibit vessel sprouting and growth in various tumor models^10^. Given the anti-angiogenic effects of RA, we speculate that RA is a potential agent for the treatment of CNV.

Herein, we designed a novel retinoic acid drug, EYE-502, and investigated its therapeutic effects on CNV formation. The results showed that EYE-502 could reduce the area of CNV and decrease pericyte coverage. EYE-502 exerted its anti-angiogenic effect by simultaneously antagonizing Wnt/β-catenin pathway in ECs and PDGF/PDGFRβ/PI3K/Akt signaling in pericytes, which may embody potential implications for the treatment in diseases related to CNV.

## Materials and methods

### Animals

C57BL/6 mice were purchased from Nanjing Qinglongshan Experimental Animal Center (Nanjing, China). All treatments were authorized by the Animal Ethics Committee of Nanjing Medical University and performed in accordance with the Association for Research in Vision and Ophthalmology (ARVO) Statement for the Use of Animals in Ophthalmic and Vision Research. The animals were maintained with free access to food and water on a 12 h light/dark cycle. The mice were euthanized by 5% pharmaceutical grade isoflurane inhalation. All experiments were carried out in compliance with the ARRIVE guidelines (https://arriveguidelines.org) and all methods were performed according to relevant guidelines and regulations.

### Cell culture

Human pericytes were obtained from Cell Systems Corporation (USA). They were cultured with Dulbecco's modified Eagle's medium (DMEM; 8120034, Gibco, USA) supplemented with 10% fetal bovine serum (FBS; 16140071, Gibco, USA) and 1% penicillin–streptomycin (15140122, Gibco, USA) at 37 °C in a humidified atmosphere of 95% air and 5% CO_2_.

### Primary isolation of choroidal EC and pericyte and culture

Primary choroidal endothelial cells (CECs) and pericytes were isolated from C57BL/6 mice (4–6 weeks old) as shown in the previous studies^11,12^. Briefly, choroidal tissues were digested with 0.05% trypsin (25300120, Gibco, USA) at 37 °C for 30 min. After removing RPE layers, they were digested in 5 ml of digestion solution containing Collagenase II (200 U/ml, 2275MG100, BioFroxx, China) and DNase I (30 U/mL, D7073, Biosharp, China) at 37 °C for 1 h. Then, the cell mixtures were filtered using a 70-μm filter (BS-70-XBS, Biosharp, China). CECs were isolated using anti-CD31 antibody-coated Dynabeads (Invitrogen, USA). The freshly isolated CECs were cultured on collagen IV (17104019, Gibco, USA) coated 6-well plates in the Microvascular Endothelial Growth Medium (EGM2-MV; CC-3202, Lonza) at 37 °C in a humidified atmosphere of 5% CO_2_. Primary pericytes were isolated using anti-PDGFRβ antibody-coated Dynabeads (Invitrogen, USA) and cultured in DMEM with 10% FBS at 37 °C and 5% CO_2_. CECs and pericytes within the three passages were used for experiments.

### MTT assay

Cell viability was measured by 3-(4, 5-dimethylthiazol-2-yl)-2, 5-diphenyl-tetrazolium-bromide (MTT) assay. 1 × 10 ^4^ cells per well of CECs or pericytes were planted onto 96-well plates. Following the required treatment, the medium was removed and the cells were incubated with 100 μl of MTT solution (5 mg/ml, 3580GR001, Biofroxx, China) at 37 °C for 3 h. Subsequently, the MTT solution was removed and the formazan crystals were dissolved in 100 μl of DMSO. After incubation on a shaker for 10 min, the absorbance of each well was measured using a microplate reader at 570 nm wavelength (Molecular Devices, USA).

### Western blot

Total proteins were obtained by incubating cells or tissues with the cold radioimmunoprecipitation assay (RIPA) lysis buffer (P0013B, Beyotime, China) supplemented with the protease inhibitor cocktails (Roche, Basel, Switzerland). Specifically, the tissues were shattered by ultrasound and incubated with RIPA, which the cells were lysed directly with RIPA. The lysates were centrifuged at 12,000×*g* for 30 min at 4 °C to collect the supernatants. The protein concentrations in the supernatant were determined by the BCA Protein Assay Kit (Pierce, Appleton, WI). Equal amounts of protein samples were subjected for the denaturing SDS–polyacrylamide gels and transferred onto the immobilon-polyvinylidene fluoride (PVDF) membranes (Merck Millipore, Billerica, MA, USA) by electroblotting. The membranes were blocked with 5% BSA in 0.05% Tween-20 TBST for 30 min at room temperature and then incubated with the primary antibodies overnight at 4 °C. On the second day, after washing with TBST and incubating with the secondary antibodies for 2 h at room temperature, the protein signals were visualized by the enhanced chemiluminescence method. Image J software was used to quantify the amount of protein expression.

### EdU assay

Cell proliferation was determined through 5‐ethynyl‐2′‐deoxyuridine (EdU) staining using the BeyoClick EdU Cell Proliferation Kit (C0071S, Beyotime, China). According to the manufacturer’s instruction, the cells were incubated with 10 μM of EdU for 2 h after the required treatment. Then, these cells were fixed, permeabilized, and stained with azide dye solution for 30 min. The nuclei were stained with DAPI (C1002, Beyotime, China) and visualized under an IX73P1F fluorescence microscope (Olympus, Tokyo, Japan). Image J software was used to quantify the proportion of EdU-positive cells.

### Tube formation assay

The individual wells of a 24-well plate were coated with 50 µL of the pre-cooled Matrigel (356230, Corning, USA) and placed in an incubator at 37 °C for 30 min. After the required treatment, ECs were harvested, re-suspended in the conditioned medium, and seeded onto the solidified gel (3 × 10^5^ cells per well) for 6 h. Tube formation was captured by an IX73P1F fluorescent microscope (Olympus, Tokyo, Japan). Image J software was used to calculate the tube length to assess the ability of tube formation.

### Cell migration assay

Transwell chamber with 8.0 µm pore membrane (Corning, USA) was used to detect cell migration ability. Briefly, ECs or pericytes (1 × 10^5^ cells per well) suspended in the serum-free medium were seeded onto the upper chambers. The lower chambers were added with the complete medium as the chemoattractant. After 12 h culture, the cells on the polycarbonate membranes were fixed with methanol for 15 min and stained with 0.5% crystal violet (C805211, Macklin, China) for 15 min at room temperature. The remaining cells on the upper surface of membranes were carefully removed with a cotton swab. Image J software was used to evaluated cell migration ability.

### ECs and pericytes co-culture assay

The mixtures of CECs and pericytes (at a ratio of 1:1) were suspended in the complete medium and seeded onto the Matrigel-precoated 24-well plate, then incubated at 37 °C in a humidified atmosphere of 5% CO_2_ for up to 12 h. The harvested cells were fixed in 4% paraformaldehyde for 15 min, washed with PBS, and permeabilized with PBS containing 0.1% Triton X-100 for 30 min. These cells were stained with CD31 (1:200; 550300, BD Pharmingen) and NG2 (1:100; ab50009, Abcam) to identify ECs and pericytes, respectively. The images were observed under an IX73P1F fluorescent microscope (Olympus, Tokyo, Japan). Image J software was used to calculate pericyte coverage.

### Annexin V-FITC/PI apoptosis assay

Cell apoptosis was detected using the Annexin V-FITC/PI Apoptosis Detection Kit (Vazyme, A211-01, China). ECs or pericytes were washed once with PBS, trypsinized, and resuspended in the medium. 1 × 10^5^ of cells were centrifuged. The cell pellets were washed with PBS, resuspended in 100 μl of binding buffer, and incubated with 5 μl of FITC Annexin V and 5 μl of PI for 10 min. Finally, all samples were analyzed by flow cytometry (BD Biosciences, San Diego, CA, USA). The graph was plotted using FlowJo 7.6.5 software (FLOWJOLLC, Ashland, KY, USA).

### Choroid sprouting assay in vitro

The eyes were enucleated from 3-week-old C57BL/6 mice and kept in ice-cold DMEM. The choroidal complex was isolated and cut into small pieces of approximately 1 × 1 mm^2^. The pieces were embedded on ice in 50 µL of growth factor-reduced Matrigel in a 24-well plate (day 0). The Matrigel was polymerized for 30 min at 37 °C and overlaid with DMEM supplemented with 10% serum. Choroidal sprouting was observed on day 3 to day 5 post-culture after the required treatment. To analyze the choroid sprouting area, the images were taken at 4 × magnification and the regrowth of microtubules was quantified by Image J software.

### Quantification of pericyte coverage

The sprouting choroidal tissue was used for detecting pericyte coverage by immunofluorescence staining. The choroidal explants were gently washed with ice-cold PBS, fixed with 4% paraformaldehyde for 15 min at room temperature, and blocked with 0.1% Triton X-100/3% BSA for 30 min at 37 °C. Pericytes were labeled with NG2 (1:100; ab50009, Abcam) overnight at 4 °C and Alexa Fluor 594 goat anti-mouse IgG (1:500; A11005, Invitrogen) for 3 h at room temperature. ECs were labeled with Isolectin GS-IB4 (1:100; L2895, Sigma, USA) for 2 h at room temperature. The images were captured under a fluorescence microscope (Olympus, Tokyo, Japan). NG2 positive area was measured using the Image J software.

### Laser-induced choroidal neovascularization

C57BL/6J mice (8-weeks old) were anesthetized with an intraperitoneal injection of 100 mg/kg of ketamine and 20 mg/kg of xylazine. The pupils were dilated using 1% pharmaceutical tropicamide. Using the OcuLight GLx Laser System (Iridex, USA), four laser burns were induced at 3, 6, 9, and 12 o’clock positions around the optic nerve. Laser parameters were 532 nm wavelength, 50 μm spot size, 100 mW power, and 100 ms duration. Only the burns that produced a bubble at the time of laser photocoagulation were included in this study. Laser spots containing hemorrhage at the laser site were excluded from subsequent analysis. These mice received EYE-502 monotherapy treatment immediately after laser photocoagulation. On day 14, the eyes were removed and processed for immunostaining. RPE-choroid complexes were incubated with NG2 at 4 °C overnight, followed by staining with Alexa Fluor 594 goat anti-mouse IgG for 3 h at room temperature in the dark to label pericytes. Isolectin GS-IB4 staining was conducted for 2 h to identify ECs at room temperature.

### Intravitreal injection

C57BL/6 mice were divided randomly into different experimental groups. Intravitreal injection was conducted using PBS (Ctrl), 0.1% DMSO (vehicle control), EYE-502, or Aflibercept in each experimental group. About 2 μl of drugs were delivered intravitreally using a Hamilton syringe equipped with a 30-gauge needle (Reno, NV, USA). The ocular surface was covered with levofloxacin hydrochloride eye gel. The mice were kept on the heating pads until recovery from anesthesia.

### Hematoxylin and eosin (H&E) staining

The enucleated eyes were fixed in 4% paraformaldehyde (PFA) for 24 h at 4 °C. Then, the eyes were dehydrated with a graded series of ethanol and embedded in paraffin. The samples were cut through the optic nerve head vertically to a thickness of 5 μm. The paraffin-embedded tissue sections were dewaxed with xylene and rehydrated in the successive ethanol baths. The sections were stained with hematoxylin for 1.5 min, washed 3 times with the double-distilled water, and stained with eosin for 50 s. Finally, the tissues were dehydrated with 100% ethanol, cleared in xylene, and fixed with neutral resin. The images were taken from the optic nerve approximately 2–3 disc-diameters.

### TUNEL staining

The apoptosis of ocular tissues were detected by TUNEL assays using the In Situ Cell Detection Kit (C1086, Beyotime, China). Briefly, the paraffin-embedded tissue sections were dewaxed with xylene and rehydrated using a graded series of alcohol and distilled water. The sections were incubated in the permeabilization solution (20 μg/ml proteinase K in 10 mM Tris/HCl) at room temperature for 15 min and washed with distilled water. Then, 50 μl of TUNEL reaction mixtures were added and incubated for 1 h at 37 °C in the dark. Finally, the nuclei were counterstained with DAPI and the slides were observed under a fluorescence microscope (Olympus, Tokyo, Japan). For the quantification, TUNEL-positive cells were counted in 10 randomly selected fields (× 200 magnification) per section.

### Electroretinography (ERG)

The single-bright flash ERG was recorded using the custom DTL fiber electrodes with an Espion testing system and ColorDome LED/Xenon-full field stimulator (Diagnosys LLC, Lowell, MA). Following the required treatment, the mice were anesthetized after dark adaptation overnight. After the pupils were dilated with 1% pharmaceutical tropicamide, the gold loop electrode was placed in the center of the cornea. The reference electrode was placed hypodermically in the cheek and the grounding electrode was attached subcutaneously near to the tail. Band-pass filter cutoff frequencies were 0.3 and 500 Hz for all flash ERG. Each record represents an average of 2–6 responses. The a-wave (first negative peak) and b-wave (first positive peak) amplitudes were measured and recorded.

### Network pharmacology analysis

EYE-502 was transformed into the standard canonical smiles format “CC1(C)CCC(C)(C)C2=C1C=CC(NC(=O)C1=CN=C(N=C1)C(O)=O)=C2” and imported into the SuperPred database (https://prediction.charite.de/) and Swiss target prediction database (https://swisstargetprediction.ch/) to predict the targets of EYE-502. The keyword “choroidal neovascularization” was inserted into the GeneCards database (https://www.genecards.org/) to search for the genes potentially involved in the pathogenesis of CNV. The additive results of SuperPred database and Swiss target prediction database was used as the predicted targets of EYE-502, and then intersected with CNV-related genes to obtain the overlapped genes. The overlapped genes were used for KEGG pathway analysis and GO analysis. The string planform (https://cn.string-db.org/) was used to conduct Protein–Protein Interaction (PPI) analysis and PPI network was visualized by Cytoscape 3.9.2 software.

### Quantitative real-time PCR

Total RNAs were extracted using TRIzol reagent (15596026, Invitrogen, USA) and reversely transcribed with HiScript III RT SuperMix (R323, Vazyme, China). GAPDH was detected as an internal control to normalize target gene expression. The primers used were shown as follows: Cyclin D1: forward 5′-GCT GCG AAG TGG AAA CCA TC-3′, reverse 5′-CCT CCT TCT GCA CAC ATT TGA A-3′; c-Myc: forward 5′-ATG GCC CAT TAC AAA GCC G-3′, reverse 5′-TTT CTG GAG TAG CAG CTC CTA A-3′; PPARδ: forward 5′-ACA GTG ACC TGG CGC TCT TC-3′, reverse 5′-CAG GCT TGC TGA ACG TGA AG-3′; PDGF-B: forward 5′-CTC GAT CCG CTC CTT TGA TGA-3′, reverse 5′-CGT TGG TGC GGT CTA TGA G-3′; GAPDH: forward 5′-AGG TCG GTG TGA ACG GAT TTG-3′, reserve 5′-GGG GTC GTT GAT GGC AA CA-3′. The 2^−ΔΔCt^ method was used for quantitative analysis of gene expression.

### Statistical analysis

The normality of data and homogeneity of variances were tested by Shapiro–Wilk and Levene tests, respectively. All continuous data were expressed as mean ± SEM. Student's *t* test or one-way analysis of variance (ANOVA) followed by Bonferroni’s post hoc test were applied for the comparison of normally distributed data with equal variance. Mann–Whitney *U* test or Kruskal–Wallis test followed by Dunn’s post-correction test were applied for nonparametric or non-normally distributed data. *P* < 0.05 was considered statistically significance and analyzed using GraphPad Prism 8. For in vivo data, each *n* value corresponds to a single mouse. For in vitro data, each *n* value corresponds to an independent experiment.

## Results

### Administration of EYE-502 has no detectable cytotoxicity and tissue toxicity

EYE-502 was a novel RA durg designed based on tazarotene (Fig. S1). Tazarotene is a receptor-selective aromatic RA that binds to the RAR and exerts biological effects by regulating gene transcription^13^. The structure of EYE-502 confers better water solubility. Toxicity of molecules is a very important part for drug development.

**Figure 1:**
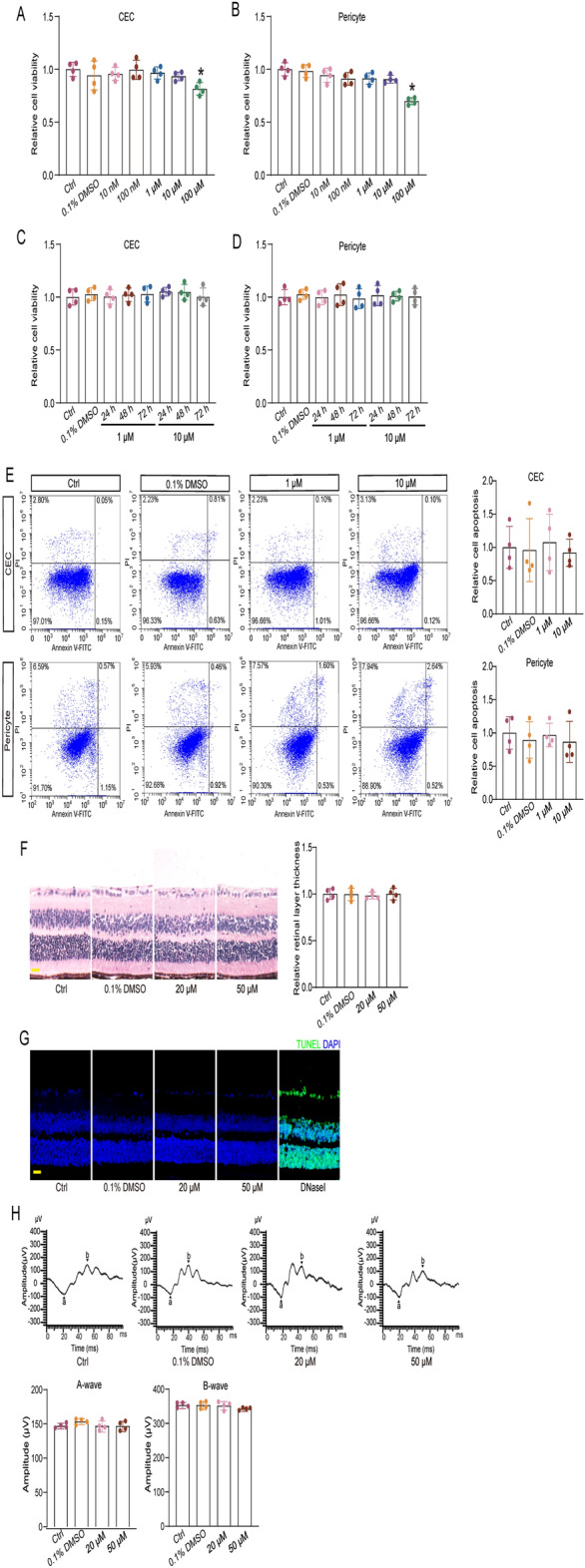
Administration of EYE-502 has no detectable cytotoxicity and tissue toxicity. (**A**,**B**) CECs and pericytes were incubated with the tested concentrations of EYE-502 or left untreated (Ctrl) for 24 h. Cell viability was detected by MTT assay (n = 4). (**C**,**D**) CECs and pericytes were incubated with EYE-502 (1 μM and 10 μM) for 24 h, 48 h, or 72 h. Cell viability was detected by MTT assay (n = 4). (**E**) Cell apoptosis was determined by Calcein-AM/PI staining and Annexin/PI flow cytometry. Scale bar, 20 μm (n = 4). (**F**) The eyes were received intravitreal injections of PBS (Ctrl), 0.1% DMSO, or EYE-502 for 7 days. Hematoxylin and eosin (H&E) staining was conducted to detect the change of retinal histological structure. Scale bar, 50 μm (n = 4). (**G**) TUNEL staining was conducted to detect retinal apoptosis. Scale bar, 50 μm (n = 4). (**H**) Electrophysiology assays were conducted to detect retinal visual function. Amplitudes of A and B waves were statistically analyzed (n = 4). *P < 0.05 versus Ctrl group; One-way ANOVA followed by the post hoc Bonferroni test.

We first used choroidal ECs (CECs) and retinal pericytes to investigate the cytotoxicity of EYE-502 in vitro. CECs or pericytes were treated with different concentrations of EYE-502 for 24 h. MTT assays showed that administration of EYE-502 did not cause any cytotoxic effect on CECs and pericytes at concentrations ranging from 10 nM to 10 μM. However, cell viability was significantly reduced when the concentration of EYE-502 was greater than 10 μM (Fig. 1A,B). Moreover, administration of EYE-502 did not cause significant toxicity on CECs or pericytes at 1 μM or 10 μM for 48 h and 72 h (Fig. 1C, D). Annexin/PI flow cytometry showed that EYE-502 did not induce the apoptosis of CECs and pericytes at the concentrations lower than 10 μM (Fig. 1E).

We then investigated whether administration of EYE-502 had the potential tissue toxicity in vivo. The retinas received an intravitreal injection of PBS (Ctrl group), 0.1% DMSO or EYE-502 (20 μM or 50 μM) for 7 days. Hematoxylin and eosin (H&E) staining revealed that administration of EYE-502 did not cause any obvious changes in the thickness and structure of retinal layers (Fig. 1F). TUNEL assays showed that there was no obvious apoptosis in retinal cells in EYE-502-treated group (Fig. 1G). Electrophysiological tests showed that there was no significant change in the A- and B- wave amplitudes in the EYE-502 group compared with the control group, indicating that EYE-502 treatment did not impair retinal visual function (Fig. 1H).

### EYE-502 regulates choroidal EC function in vitro

**Figure 2:**
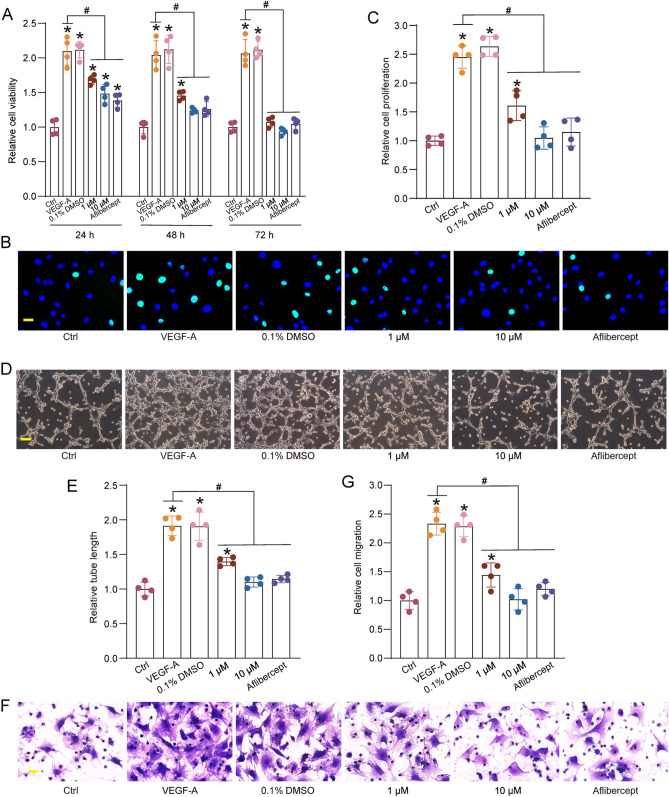
EYE-502 regulates choroidal EC function in vitro. (**A**) CECs were exposed to VEGF (10 ng/ml), VEGF plus 0.1% DMSO, VEGF plus EYE-502 (1 μM or 10 μM), or aflibercept for 24 h, 48 h, or 72 h. The untreated group was taken as the Ctrl group. Cell viability was detected by MTT assays (n = 4). (**B**–**F**) CECs were exposed to VEGF (10 ng/ml), VEGF plus 0.1% DMSO, VEGF plus EYE-502 (1 μM or 10 μM), or aflibercept for 24 h. The untreated group was taken as the Ctrl group. EdU incorporation assays were performed to detect cell proliferation and the EdU-positive cells were quantitated. DAPI, blue; EdU, green. Scale bar, 20 μm (**B**,**C**; n = 4). Matrigel-based tube formation assays were conducted to evaluate the formation of new vessels. Scale bar, 100 μm (**D**,**E**; n = 4). Transwell assays were conducted to determine cell migration abilities. Scale bar, 20 μm (**F**,**G**; n = 4). *P < 0.05 versus Ctrl group; ^#^*P* < 0.05 versus VEGF group; One-way ANOVA followed by Bonferroni test.

VEGF is a powerful pro-angiogenic factor that can increase vascular permeability and promote EC proliferation, migration, and tube formation^14^. We pretreated CECs with VEGF to mimic EC activation during pathological angiogenesis and investigated whether administration of EYE-502 had an anti-angiogenic role on CECs. MTT assays showed that VEGF treatment led to increased viability of CECs. The increase in CEC viability was markedly reduced following the administration of EYE-502, showing a similar effect as Aflibercept (Fig. 2A). 5‐ethynyl‐2′‐deoxyuridine (EdU) assays and transwell assays showed that administration of EYE-502 significantly reduced the proliferation ability and migration ability of CECs induced by VEGF (Fig. 2B–E). Matrigel tube formation assays showed that administration of EYE-502 significantly reduced the tube formation ability of CECs induced by VEGF (Fig. 2F,G). Moreover, administration of EYE-502 had a similar anti-angiogenic effects as aflibercept (Fig. 2B–G). Collectively, these results suggest that administration of EYE-502 inhibits VEGF-induced EC activation in vitro.

### EYE-502 regulates pericyte function in vitro

**Figure 3:**
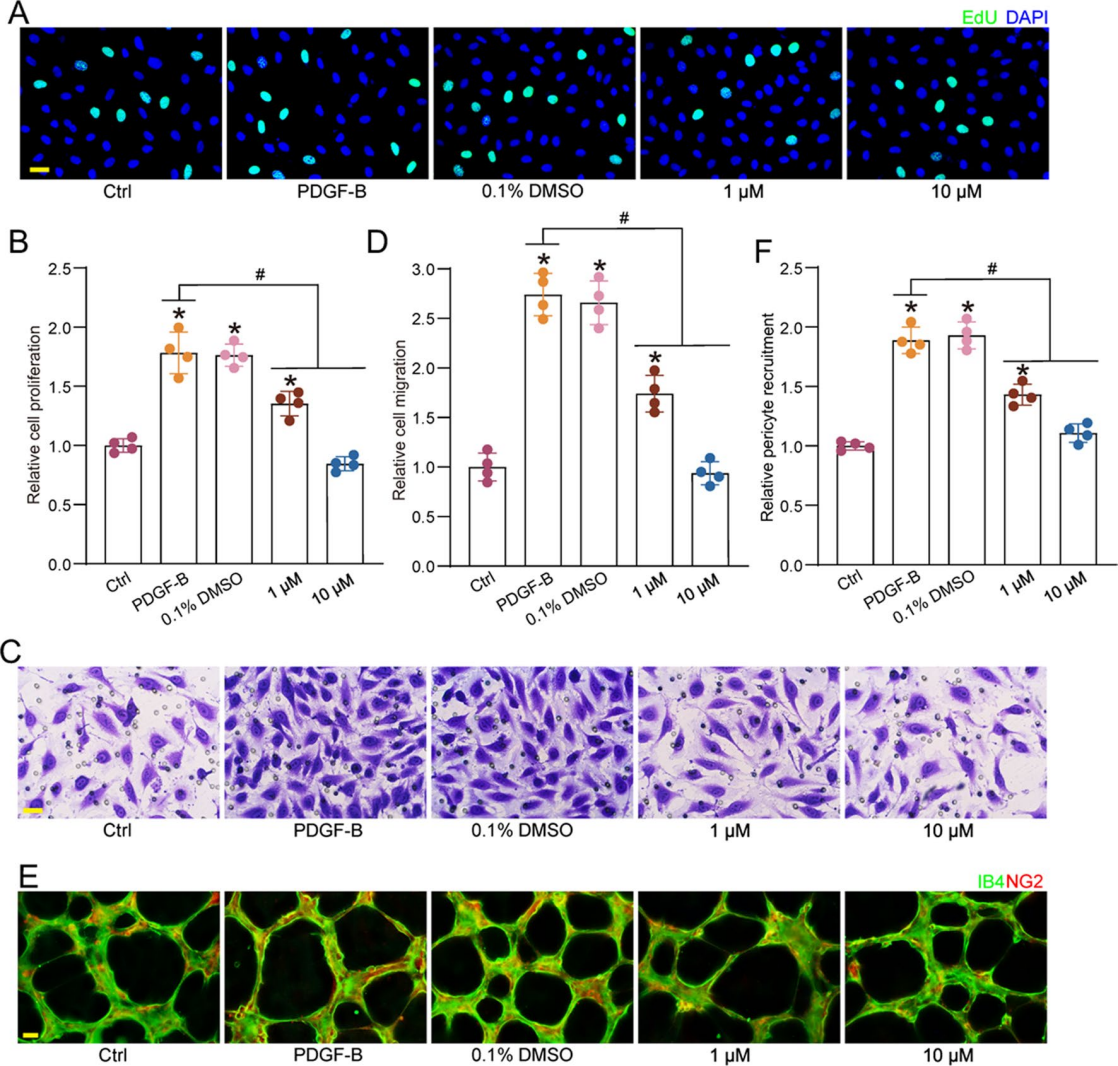
EYE-502 regulates choroidal pericyte function in vitro. Pericytes were pretreated with PDGF-B (25 ng/ml), then treated with 0.1 DMSO, EYE-502 (1 μM and 10 μM) or aflibercept for 24 h. The group untreated was taken as the Ctrl group. (**A**,**B**) Cell proliferation was detected by EdU staining. Scale bar, 20 μm (n = 4). (**C**,**D**) Transwell assays were conducted to determine cell migration abilities. Scale bar, 20 μm (n = 4). (**E**,**F**) Pericytes were co-cultured with CECs on the matrigel matrix for 12 h and then stained with NG2 (pericytes) and CD31 (CECs) to detect the recruitment of pericytes toward CECs. Scale bar, 50 μm (n = 4). *P < 0.05 versus Ctrl group; ^#^*P* < 0.05 versus PDGF-B group; One-way ANOVA followed by Bonferroni test.

PDGF-B is the most important growth factor for maintaining pericyte survival, which can inhibit pericyte apoptosis, induce pericyte proliferation and migration, and recruit pericytes to synergize with ECs to form the stable blood vessels^15^. We subsequently explored whether administration of EYE-502 could regulate PDGF-B-induced pericyte activation in vitro. EdU assays showed that administration of EYE-502 could inhibit PDGF-B-induced pericyte proliferation (Fig. 3A,B). Transwell assays showed that administration of EYE-502 could inhibit PDGF-B-induced pericyte migration (Fig. 3C,D). Matrigel co-culture assays showed that administration of EYE-502 significantly reduced the number of pericytes recruited to ECs (Fig. 3E,F). Primary choroidal pericytes were isolated to detect the effects of EYE-502 administration on choroidal pericyte function in vitro. The results indicated that administration of EYE-502 could decrease the proliferation and migration ability of choroidal pericytes and reduce the recruitment ability of choroidal pericytes to CECs (Fig. S2). These results suggest that administration of EYE-502 can inhibit PDGF-B-induced pericyte hyper-activation in vitro.

### EYE-502 suppresses choroidal vascular sprouting and pericyte coverage

**Figure 4:**
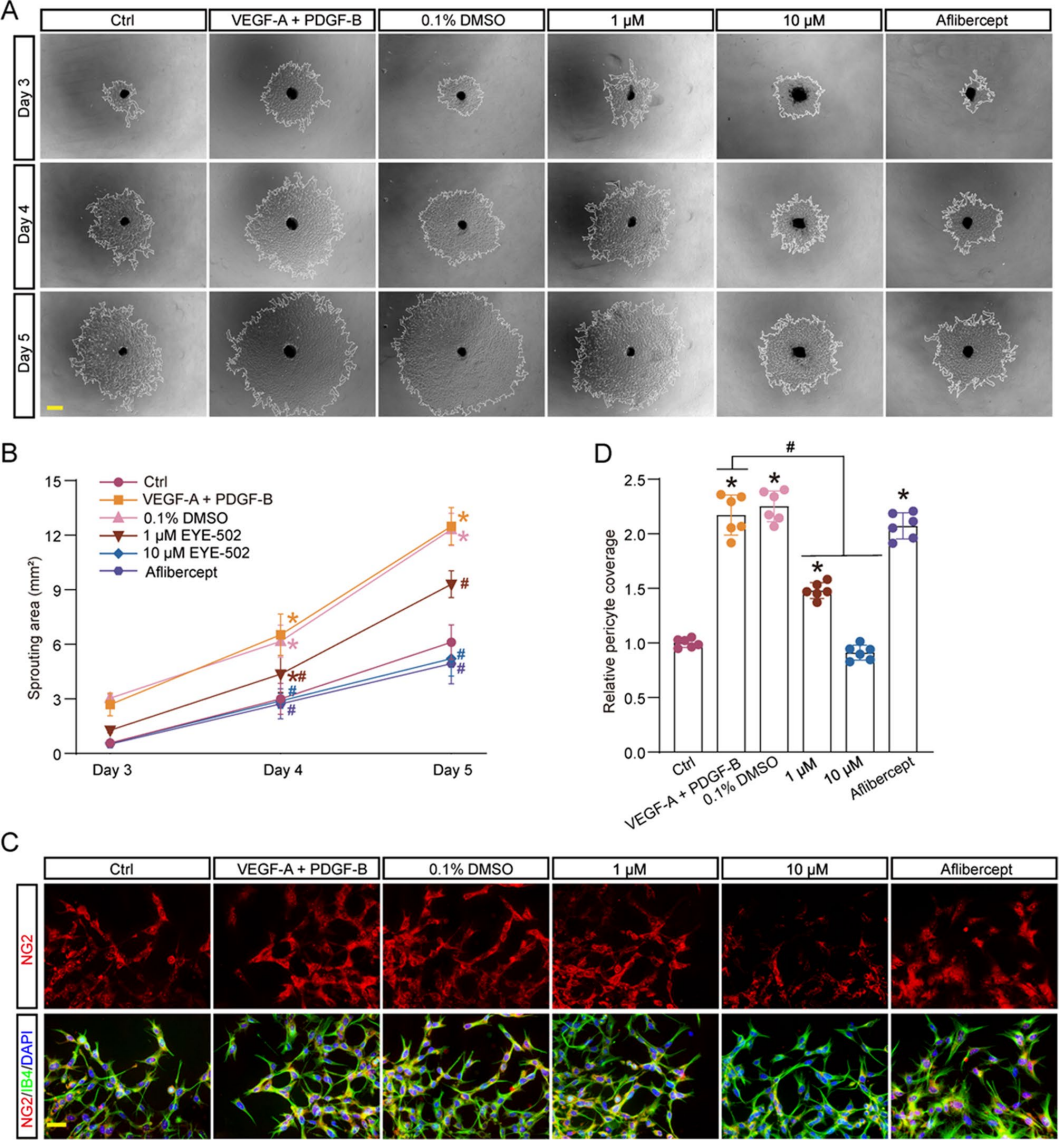
EYE-502 suppresses choroidal vascular sprouting and pericyte coverage. (**A**,**B**) The choroid explants were incubated with 0.1% DMSO, EYE-502 (1 μM and 10 μM), or aflibercept. The untreated group was taken as the Ctrl group. Choroid sprouting was measured using an inverted microscope and expressed as relative changes compared with the control group. Scale bar, 200 µm (n = 6). (**C**,**D**) The extending growth cones were stained with GS-IB4 (ECs) and NG2 (pericytes). Scale bar, 20 μm (n = 6). *P < 0.05 versus the Ctrl group; ^#^*P* < 0.05 versus VEGF-A + PDGF-B group; Kruskal–Wallis’s test followed by Bonferroni’s post hoc test.

Choroidal vascular sprouting can be used to study the growth of choroidal vessels in vitro^16^. Following Matrigel solidified, the choroidal explants were incubated with the complete medium containing VEGF-A and PDGF-B, then added 0.1% DMSO, EYE-502 (1 μM and 10 μM), or aflibercept for 5 days. Choroidal sprouting was observed from day 3 to day 5. The results showed that the sprouting area of EYE-502 group and aflibercept group was significantly reduced compared to VEGF-A plus PDGF-B group or 0.1% DMSO group (Fig. 4A,B).

Choroidal sprout is a tubular growth composed of GS-IB4^+^ ECs surrounded by NG2^+^ pericytes^17^. GS-IB4 and NG2 staining were used to label ECs and pericytes in choroidal sprouting. Administration of EYE-502 led to reduced pericyte coverage compared with VEGF-A plus PDGF-B or 0.1% DMSO group. However, pericyte coverage in aflibercept group did not alter compared with that in VEGF-A plus PDGF-B or 0.1% DMSO group (Fig. 4C,D). In addition, 10 µM EYE-502 led to a marked reduction of angiogenic area and pericyte coverage compared with 1 μM EYE-502 group, indicating that EYE-502 exerts its anti-angiogenic effects in a dose-dependent manner. Collectively, these results suggest that administration of EYE-502 can inhibit CNV formation and reduce pericyte coverage*.*

### EYE-502 suppresses CNV formation and reduces pericyte coverage

**Figure 5:**
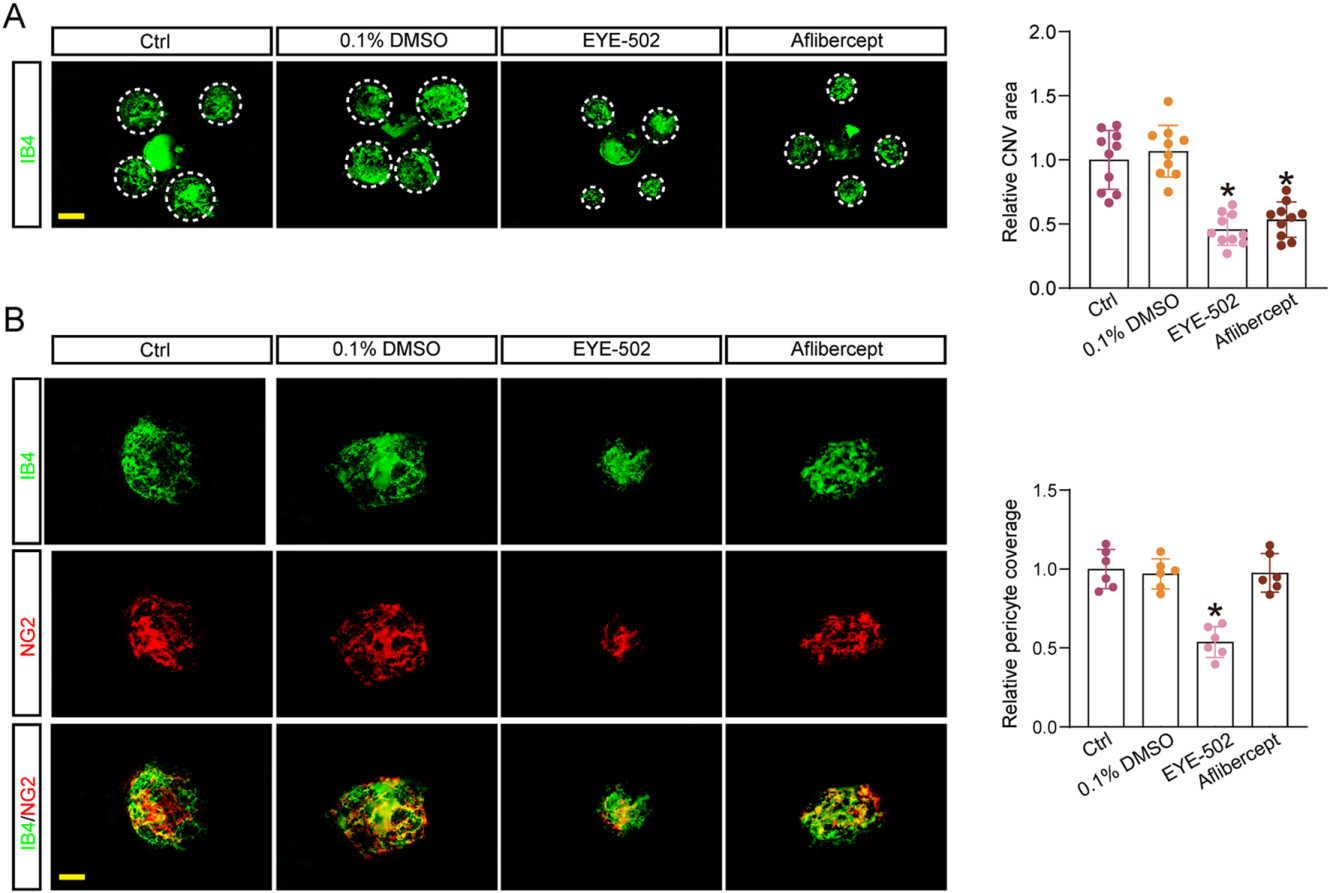
EYE-502 suppresses CNV formation and reduces pericyte coverage. (**A**,**B**) The mice received intravitreal injections of PBS, 0.1% DMSO, EYE-502 (50 μM), or aflibercept for 14 days following laser photocoagulation. The group treated with PBS was taken as the Ctrl group. RPE/choroid complexes were prepared for Isolectin-B4 staining and evaluation of CNV area. Dashed lines delineate the lesions. Scale bar, 100 μm (**A**, n = 10). Pericyte coverage was quantified by staining RPE-choroidal flat mounts with Isolectin-B4 and NG2. Scale bar, 50 μm (**B**, n = 6). *P < 0.05 versus Ctrl group; Kruskal–Wallis’s test followed by Bonferroni’s post hoc test.

We then investigated the therapeutic effects of EYE-502 on CNV formation by intravitreal injection of EYE-502. Following laser photocoagulation, each eye received 0.1% DMSO, EYE-502, aflibercept, or left untreated (control group) for 14 days and the choroid was exacted to label CNV formation by IB4 staining. The results revealed that intravitreal injection of EYE-502 or aflibercept significantly reduced CNV area compared with the control group (Fig. 5A). We also stained with IB4 and NG2 to detect pericyte coverage on CNV. The results showed that administration of EYE-502 could reduce the coverage of pericytes on CNV. By contrast, administration of aflibercept did not affect pericyte coverage on CNV (Fig. 5B).

### Prediction the target genes of EYE-502

**Figure 6:**
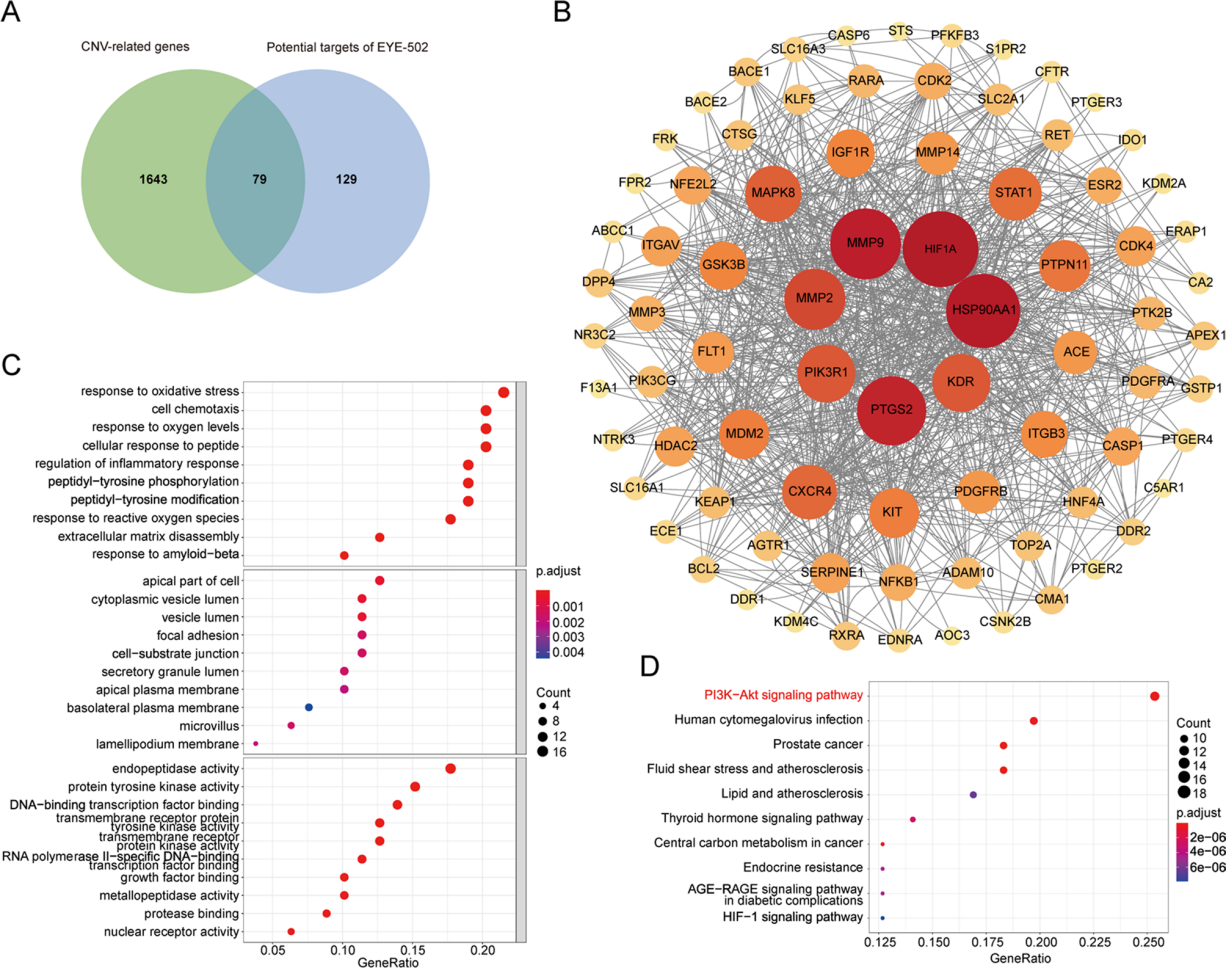
Prediction the target genes of EYE-502. (**A**) 79 genes were predicted as the target genes of EYE-502 for CNV treatment. (**B**) PPI was conducted to obtain the interaction relationship between the target proteins. (**C**) GO enrichment analysis was conducted to obtain the top ten biological processes, cellular components, and molecular functions of the target genes of EYE-502. (**D**) KEGG enrichment analysis was conducted to obtain the top ten pathways of the target genes of EYE-502.

We next searched for the potential targets and potential pathways that EYE-502 acted on CNV to understand the mechanism of drug action. From the SuperPred database and Swissstar database, 130 and 101 potential targets for EYE-502 were obtained, respectively. 208 potential targets were obtained following data fusion.The keyword “choroidal neovascularization” was inserted into the GeneCards database (https://www.genecards.org/) to search for the genes potentially involved in the pathogenesis of CNV. These genes were defined as CNV-associated genes. A list of 1723 genes related to CNV was obtained from the Genecards database. By intersecting the target genes of EYE-502 with CNV-related genes, 79 potential targets of EYE-502 were identified (Fig. 6A). According to PPI, the top 10 nodes in the ranking were HIF1A, HSP90AA1, MMP9, PTGS2, MMP2, PI3KR1, KDR, MAPK8, CXXCR4, and STAT1 (Fig. 6B). The top ten biological processes, cellular components, and molecular functions connected to the target genes of EYE-502 were shown in Fig. 6C following GO enrichment analysis. PI3K-Akt signaling pathway was ranked as the top 1 signaling pathway involved in EYE-502-medaited CNV reduction (Fig. 6D).

### RARs are primary cellular targets for EYE-502

**Figure 7:**
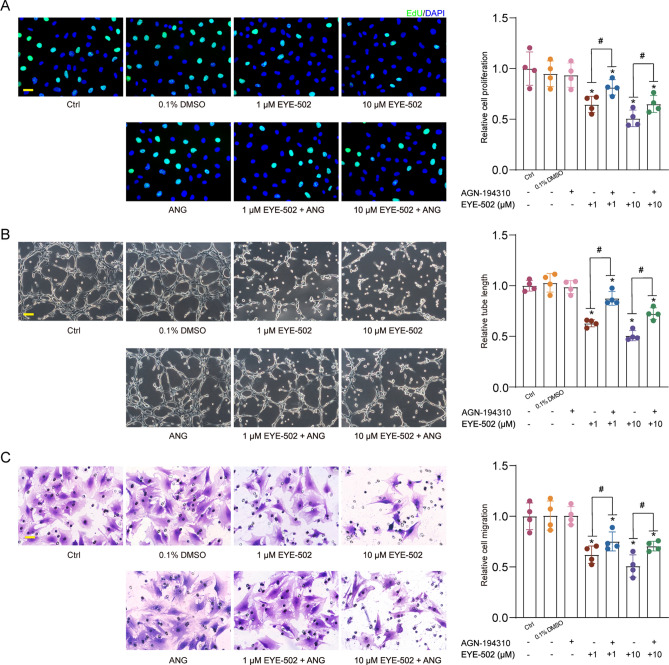
RARs are the primary cellular targets for EYE-502. (**A**–**C**) CECs were pre-treated with or without AGN-194310 for 12 h to block the activity of RARs. Then, they were exposed to 0.1% DMSO, EYE-502 (1 μM and 10 μM) for 24 h. The group untreated was taken as the Ctrl group. EdU assays were conducted to detect cell proliferation and the EdU-positive cells were quantitated. DAPI, blue; EdU, green. Scale bar, 20 μm (**A**, n = 4). Matrigel-based tube formation assays were conducted to evaluate the formation of new vessels. Scale bar, 100 μm (**B**, n = 4). Transwell assays were conducted to detect cell migration ability. Scale bar, 20 μm (**C**, n = 4). *P < 0.05 versus Ctrl group; ^#^*P* < 0.05 between the marked group; One-way ANOVA followed by Bonferroni test.

RA binds to RAR and acts as a signaling transducer by controlling the transcription of the target genes^18^. We thus investigated whether EYE-502 regulated EC function by binding to RAR. CECs were incubated with the RAR pan-antagonist, AGN-194310 to block RAR activity in CECs. The results showed that administration of EYE-502 led to decreased proliferation, migration, and tube formation ability of CECs. By contrast, pretreatment with AGN-194310 could interrupt the anti-angiogenic effects of EYE-502 on CECs as shown by enhanced proliferation, migration, and tube formation ability (Fig. 7).

RAR contains 3 subtypes, RARα, RARβ, and RARγ. We then used different RAR siRNAs to specifically silence RARα, RARβ, or RARγ in CECs or the primary choroidal pericytes. The results revealed that knockdown of RARs interrupted the anti-angiogenic effects of EYE-502 on CECs, which was similar to AGN-194310. The experiments on the primary choroidal pericytes revealed that transfection of RAR siRNAs could reserve anti-angiogenic effects of EYE-502 on pericytes, suggesting that RARs are the primary cellular target of EYE-502 (Fig. S3).

### EYE-502 exerts its anti-angiogenic effects via affecting Wnt signaling and PDGF signaling

**Figure 8:**
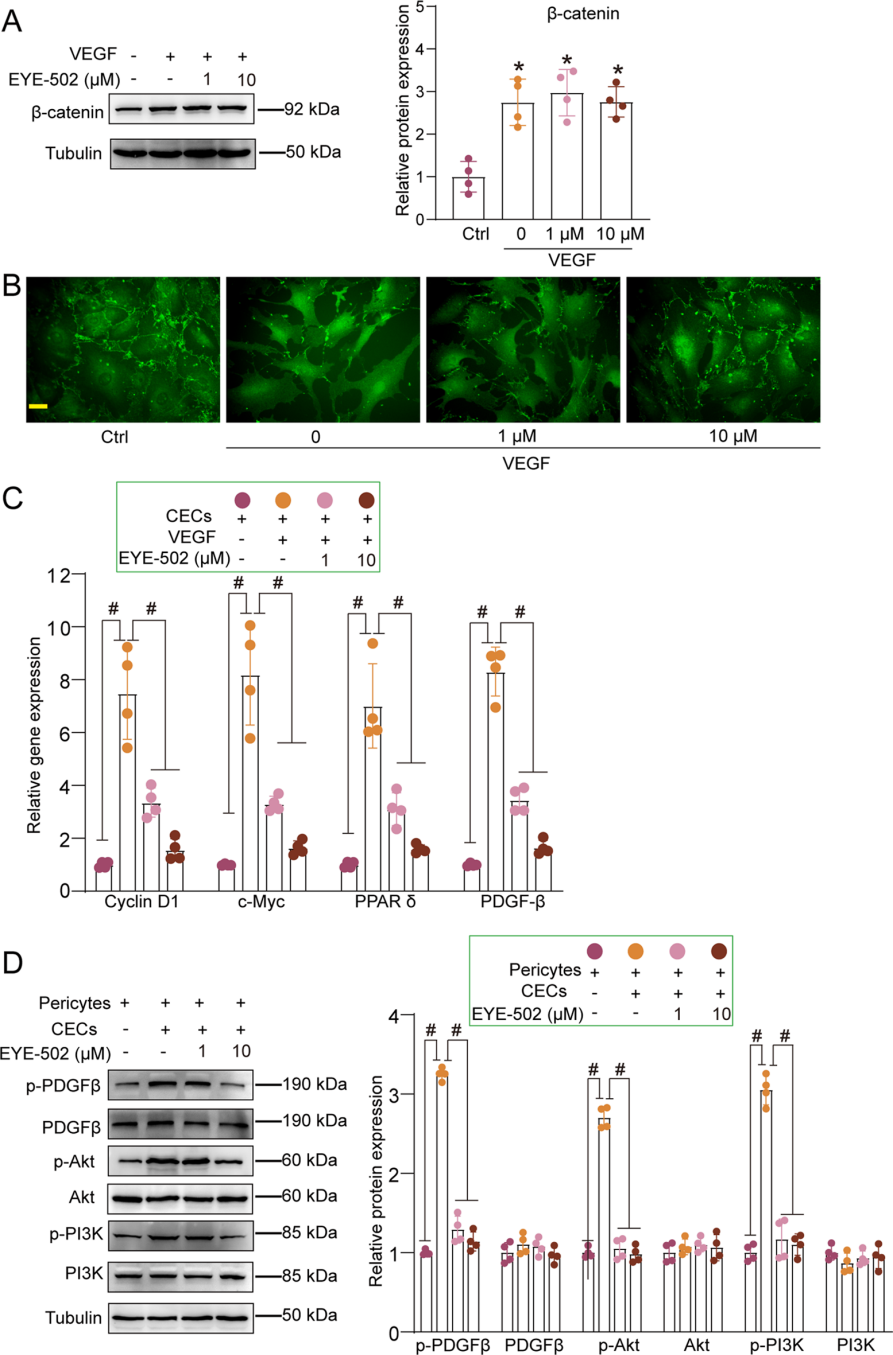
EYE-502 exerts its anti-angiogenic effects via Wnt signaling and PDGF signaling. (**A**–**C**) CECs were pretreated with VEGF (10 ng/ml), then exposed to EYE-502 (1 μM and 10 μM) for 24 h. The group without VEGF treatment was taken as the Ctrl group. Western blots were conducted to detect the expression of β-catenin (**A**, n = 4). Immunofluorescence staining was conducted to detect the levels of β-catenin expression (**B**, n = 4). Quantitative PCR assays were conducted to detect the expression of c-Myc, cyclin D1, PPAR δ, and PDGFβ (**C**, n = 4). Primary choroidal pericytes were co-cultured with or without CECs using Transwell chambers, then exposed to EYE-502 (1 μM and 10 μM) for 24 h. Western blots were conducted to detect the expression of p-PDGFRβ, PDGFRβ, p-PI3K, PI3K, p-Akt, and Akt (**D**, n = 4). *P < 0.05 versus Ctrl group; ^#^*P* < 0.05 between the marked group; One-way ANOVA followed by Bonferroni test.

Previous study has revealed that RA binds to RARα receptor in ECs and inhibits Wnt/β-catenin signaling via affecting β-catenin degradation and preventing signal transduction^19^. β-catenin can be transferred from the cytoplasm to the nucleus and trigger cell division, differentiation, and maturation^20,21^. We thus detected the levels of β-catenin expression in CECs following EYE-502 administration. Western blot analysis revealed that VEGF treatment led to increased levels of β-catenin expression, indicating that VEGF treatment led to the activation of Wnt/β-catenin signaling. However, EYE-502 treatment had no effects on the levels of β-catenin expression, suggesting that EYE-502 did not affect the stability of β-catenin in vitro (Fig. 8A). Western blot analysis showed that the levels of β-catenin expression were significantly up-regulated in CNV models, while EYE-502 administration did not affect the levels of β-catenin, suggesting that EYE-502 administration did not alter the stability of β-catenin in vivo (Fig. S4).β-catenin translocation to the nucleus is considered as another factor that affects the activation of Wnt/β-catenin signaling^22^. Immunofluorescence staining revealed that VEGF treatment accelerated the translocation of β-catenin to the nucleus, whereas EYE-502 treatment could retard β-catenin translocation (Fig. 8B). Moreover, EYE-502 treatment significantly reduced the levels of the downstream genes of β-catenin signaling, including c-Myc, cyclin D1, peroxisome proliferator-activated receptor (PPAR) δ, and PDGF-B (Fig. 8C).

Wnt/β-catenin signaling can regulate the recruitment of pericytes to the vasculature by targeting PDGF-B factor, which could bind to PDGFRβ factor expressed on the surface of pericytes^23^. PDGFRβ activates PI3K/Akt signaling to promote actin reorganization as well as cell migration and growth^24^. We thus investigated whether EYE-502 treatment could affect the activation of PDGF signaling in the primary choroidal pericytes. Western blot analysis showed that EYE-502 treatment led to decreased the levels of phosphorylated PDGFRβ (p-PDGFRβ) and the downstream signaling molecules phosphorylated PI3K (p-PI3K) and phosphorylated Akt (p-Akt) (Fig. 8D). We further used CNV models to detect whether EYE-502 inhibited CNV formation via affecting PDGF signaling in vivo. EYE-502 treatment could inhibit the upregulation of p-PDGFβ, p-PI3K, and p-Akt in CNV model (Fig. S4). Collectively, the above-mentioned results indicate that EYE-502 regulates EC and pericyte function via affecting the activation of Wnt/β-catenin signaling and PDGF signaling.

## Discussion

AMD is a multifactorial degenerative disease that can lead to great injuries on central vision. AMD can be classified into two types: neovascular AMD and non-neovascular AMD. Nearly one-third of early AMD progresses to neovascular AMD. Neovascular AMD is characterized by the formation of CNV^25^. Currently, the first-line treatment for CNV is intravitreal injection of anti-VEGF agent^26^. However, there is great burden on AMD patients due to monthly invasive injections and expensive treatment costs. Moreover, some patients showed poor responses to anti-VEGF agent^27,28^. In fact, CNV is a complex process involving multiple types of cells and signaling pathways. Thus, it is required to develop a novel method by targeting multiple vascular cells.

In this study, we designed a novel RA drug, EYE-502, and investigated its therapeutic effects on CNV formation. RA is known as a metabolite of vitamin A, which plays important roles in cell proliferation, apoptosis, and embryonic development by binding to RAR and RXR. Previous studies have reported the anti-angiogenic effects of RA^29^. RA can be used for the treatment of acute promyelocytic leukemia (APL) and plays its anti-APL effects by inhibiting mTOR signaling^30^. RA exerts its chemoprotective effect on lung cancer, breast cancer, and invasion cancer. RA treatment alters cancer proliferation and invasion by regulating the expression of RARα downstream target gene, RARβ2^31,32^. Here, we report that a novel RA drug, EYE-502, which can inhibit the formation of CNV by simultaneously targeting for ECs and pericytes. Mechanistically, EYE-502 exerts its anti-angiogenic effects by directly binding to RAR and accelerated the transfer of β-catenin into the nucleus.

ECs are the main constituent cells of blood vessels and their activation plays an important role in the process of neovascularization^33^. Elevated VEGF levels in neovascular AMD can obviously induce EC activation and proliferation, cross Bruch’s membrane and RPE layer, and ultimately develop into CNV^34^. Current treatment for CNV is mainly achieved by anti-VEGF drug. However, repeated injections may lead to impaired retinal function. EYE-502 has no obvious toxicity on ECs at the concentration up to 10 μM and has no toxicity on retinal tissue at the concentration up to 50 μM. EYE-502 could inhibit VEGF-induced dysfunction of endothelial cells (ECs) and reduce platelet-derived growth factor (PDGF)-induced recruitment of pericytes to ECs in vitro. Moreover, EYE-502 reduces the area of choroidal vascular sprouting and inhibits the formation of CNV.β-catenin is a core molecule of canonical Wnt pathway and plays an important role in cell proliferation, migration, and differentiation^35^. This study has revealed that EYE-502 has no effect on the total levels of β-catenin expression but inhibits the entry of β-catenin into the nucleus, thereby affecting cell proliferation, migration, and tube formation and inhibiting the formation of CNV. Under normal condition, β-catenin is phosphorylated by binding to the complex composed of glycogen synthase kinase 3β (GSK 3β), adenomatous polyposis coli (APC) protein, and axin^36^. Once Wnt signaling is activated, β-catenin accumulates in the cytoplasm and transfers to the nucleus, initiating the transcription of these genes, such as c-Myc, cyclinD1, peroxisome proliferator-activated receptors (PPARs)^37,38^. Numerous studies have revealed that c-Myc is widely expressed and serves as a key regulator of typical cellular processes, such as cell proliferation, differentiation, survival, and migration. c-Myc can regulate angiogenesis by promoting the production of pro-angiogenic factors^39^. Cyclin D1 is a positive regulator of cell cycle and overexpression of cyclin D1 can cause increased cell proliferation^40^. Previous study has demonstrated that cyclin D1 is essential for neovascularization^41^. PPARs are the transcription factors that are activated by ligands and subsequently bind to regulatory regions in the target genes involved in AMD. PPARs can be divided into PPARα, PPARγ, and PPARβ/δ. PPARβ/δ inhibition can efficiently reduce neovascular lesions by affecting the expression of extracellular matrix molecules and angiogenic factors^42^. Herein, administration of EYE-502 can lead to decreased levels of c-Myc, Cyclin D1, and PPARδ. EYE-502 affects the function of retinal vessel cells by repressing the transfer of β-catenin into nucleus and altering the transcription of the downstream genes, including c-Myc, cyclinD1, and PPARs.

Pericytes attached to the surface of the endothelium is another component of blood vessels. Pericytes exist at the intervals along capillary wall and are important for angiogenesis, maintenance of blood–brain barrier, and control of blood flow^43^. Pericytes loosely surround ECs to provide the conditions for EC proliferation and vessel maturation during CNV formation^44^. In addition, pericytes are known as the protective factors for ECs and increase the resistance of new blood vessels to anti-VEGF therapy^45^. These evidence suggests that pericyte is another cellular target for the treatment of CNV. EYE-502 treatment could inhibit the activation of pericytes and reduce the coverage of pericytes in CNV and reduce the resistance of new blood vessels to anti-VEGF therapy. Thus, EYE-502 could improve the therapeutic efficiency of CNV by affecting pericyte biology.

The crosstalk between ECs and pericytes is an important cause of neovascular maturation and stabilization. During the formation of CNV, ECs are activated to form neovascularization and release PDGF-B. PDGF-B is a mitogenic growth factor, which binds to PDGFRβ on the surface of pericytes and causes pericytes to proliferate, migrate, and cover the vessel wall^46^. PDGF-B plays an important role in the maturation of newly formed vessels due to the role of PDGF-B in pericyte recruitment in CNV^47^. Inhibition of PDGF-B can effectively inhibit the formation of CNV, promote the apoptosis of new blood vessels, and inhibit the expression of VEGF in ECs^48^. In this study, we show that EYE-502 not only inhibits abnormal EC activation, but also inhibits the crosstalk between ECs and pericytes by inhibiting PDGF/PDGFR signaling. PDGFR is a typical receptor tyrosine kinase (RTK) and phosphorylation of PDGFRβ initiates PDGF/PDGFRβ signaling. As a classic downstream signaling pathway of PDGFR, PI3K/Akt pathway plays an important role in multiple biological functions, such as proliferation, adhesion, migration, and invasion. Previous studies have demonstrated that PI3K/Akt pathway is important for the formation of CNV^49^. We show that EYE-502 significantly inhibits the phosphorylation of PI3K and Akt in pericytes, thereby reducing the recruitment ability and pro-angiogenic effects of pericytes, suggesting that EYE-502 affects the recruitment and the pro-angiogenic effect of pericytes via PDGF/PDGFR/PI3K/Akt signaling.

## Conclusion

This study reveals the therapeutic effect of EYE-502 on the formation of CNV. EYE-502 primarily binds to RAR, and then regulates Wnt/β-catenin pathway in ECs to suppress the proliferation, migration and tube formation of ECs. Meanwhile, EYE-502 inhibits the expression of PDGF and the downstream signal of β-catenin in ECs. Decreased PDGF secreted by ECs reduces the phosphorylation of PDGFRβ on the pericyte surface, which inhibits PI3K/Akt signaling in pericytes and ultimately prevents pericyte recruitment to retard pathological neovascular maturation. Taken together, these results suggest that EYE-502 is a new dual-pathway, dual-targeted drug for the treatment of CNV.

## Supplementary Information

Supplementary Information.

## Data Availability

Data of this study are available from the corresponding author upon reasonable request.

## References

[1] Bourne RRA (2013). Causes of vision loss worldwide, 1990–2010: A systematic analysis. Lancet Glob. Health..

[2] Mitchell P, Liew G, Gopinath B, Wong TY (2018). Age-related macular degeneration. Lancet.

[3] Ricci F (2020). Neovascular age-related macular degeneration: Therapeutic management and new-upcoming approaches. Int. J. Mol. Sci..

[4] Dorrell M, Uusitalo-Jarvinen H, Aguilar E, Friedlander M (2007). Ocular neovascularization: Basic mechanisms and therapeutic advances. Surv. Ophthalmol..

[5] Heier JS (2016). Comparison of aflibercept, bevacizumab, and ranibizumab for treatment of diabetic macular edema: Extrapolation of data to clinical practice. JAMA Ophthalmol..

[6] Wong WL (2014). Global prevalence of age-related macular degeneration and disease burden projection for 2020 and 2040: A systematic review and meta-analysis. Lancet Glob. Health.

[7] Ahmad I (2011). Regulation of ocular angiogenesis by Notch signaling: Implications in neovascular age-related macular degeneration. Invest. Ophthalmol. Vis. Sci..

[8] Browning AC, Dua HS, Amoaku WM (2008). The effects of growth factors on the proliferation and in vitro angiogenesis of human macular inner choroidal endothelial cells. Br. J. Ophthalmol..

[9] Janesick A, Wu SC, Blumberg B (2015). Retinoic acid signaling and neuronal differentiation. Cell Mol. Life Sci..

[10] Song Y, Lu H, Wang Q, Xiang R (1847). Targeting angiogenesis by blocking the ATM-SerRS-VEGFA pathway for UV-induced skin photodamage and melanoma growth. Cancers.

[11] Loeven MA (2018). A novel choroidal endothelial cell line has a decreased affinity for the age-related macular degeneration-associated complement factor H variant 402H. Invest. Ophthalmol. Vis. Sci..

[12] Zhao Z (2022). TGF-β promotes pericyte-myofibroblast transition in subretinal fibrosis through the Smad2/3 and Akt/mTOR pathways. Exp. Mol. Med..

[13] Zhang Y (2022). Synthetic retinoid kills drug-resistant cancer stem cells via inducing RARγ-translocation-mediated tension reduction and chromatin decondensation. Adv. Sci..

[14] Kase S (2010). AlphaB-crystallin regulation of angiogenesis by modulation of VEGF. Blood.

[15] Kemp SS, Aguera KN, Cha B, Davis GE (2020). Defining endothelial cell-derived factors that promote pericyte recruitment and capillary network assembly. Arterioscler. Thromb. Vasc. Biol..

[16] Basavarajappa HD (2017). Ferrochelatase is a therapeutic target for ocular neovascularization. EMBO Mol. Med..

[17] Andre H, Tunik S, Aronsson M, Kvanta A (2015). Hypoxia-inducible factor-1alpha is associated with sprouting angiogenesis in the murine laser-induced choroidal neovascularization model. Invest. Ophthalmol. Vis. Sci..

[18] Thangavelu G (2022). Retinoic acid signaling acts as a rheostat to balance Treg function. Cell Mol. Immunol..

[19] Lim YC, Kang HJ, Kim YS, Choi EC (2012). All-trans-retinoic acid inhibits growth of head and neck cancer stem cells by suppression of Wnt/β-catenin pathway. Eur. J. Cancer.

[20] Wang G (2019). Novel long noncoding RNA OTUD6B-AS1 indicates poor prognosis and inhibits clear cell renal cell carcinoma proliferation via the Wnt/β-catenin signaling pathway. Mol. Cancer.

[21] Mazumdar J (2010). O_2_ regulates stem cells through Wnt/β-catenin signalling. Nat. Cell Biol..

[22] Tian M (2020). IRF3 prevents colorectal tumorigenesis via inhibiting the nuclear translocation of β-catenin. Nat. Commun..

[23] Reis M (2012). Endothelial Wnt/β-catenin signaling inhibits glioma angiogenesis and normalizes tumor blood vessels by inducing PDGF-B expression. J. Exp. Med..

[24] Mellgren AM (2008). Platelet-derived growth factor receptor beta signaling is required for efficient epicardial cell migration and development of two distinct coronary vascular smooth muscle cell populations. Circ. Res..

[25] Kim J (2019). Tie2 activation promotes choriocapillary regeneration for alleviating neovascular age-related macular degeneration. Sci. Adv..

[26] Zhu Y, Zhang T, Xu G, Peng L (2016). Anti-vascular endothelial growth factor for choroidal neovascularisation in people with pathological myopia. Cochrane Database Syst. Rev..

[27] Bucher F (2017). Antibody-mediated inhibition of Tspan12 ameliorates vasoproliferative retinopathy through suppression of β-catenin signaling. Circulation.

[28] Beier C, Palanker D, Sher A (2018). Stereotyped synaptic connectivity is restored during circuit repair in the adult mammalian retina. Curr. Biol..

[29] Yang Z (2022). Retinoic acid inhibits the angiogenesis of human embryonic stem cell-derived endothelial cells by activating FBP1-mediated gluconeogenesis. Stem Cell Res. Ther..

[30] Liang C (2021). Overview of all-trans-retinoic acid (ATRA) and its analogues: Structures, activities, and mechanisms in acute promyelocytic leukaemia. Eur. J. Med. Chem..

[31] Ni X, Hu G, Cai X (2019). The success and the challenge of all-trans retinoic acid in the treatment of cancer. Crit. Rev. Food Sci. Nutr..

[32] Lokman NA (2019). Anti-tumour effects of all-trans retinoid acid on serous ovarian cancer. J. Exp. Clin. Cancer Res..

[33] Sluimer JC (2009). Thin-walled microvessels in human coronary atherosclerotic plaques show incomplete endothelial junctions relevance of compromised structural integrity for intraplaque microvascular leakage. J. Am. Coll. Cardiol..

[34] Zhao M (2019). Mineralocorticoid receptor antagonism limits experimental choroidal neovascularization and structural changes associated with neovascular age-related macular degeneration. Nat. Commun..

[35] Murgan S (2015). Atypical transcriptional activation by TCF via a zic transcription factor in *C.*
*elegans* neuronal precursors. Dev. Cell..

[36] Riley RS, Day ES (2017). Frizzled7 antibody-functionalized nanoshells enable multivalent binding for Wnt signaling inhibition in triple negative breast cancer cells. Small.

[37] Kalra H (2019). Extracellular vesicles containing oncogenic mutant β-catenin activate Wnt signalling pathway in the recipient cells. J. Extracell. Vesicles.

[38] Yan W (2020). N-cadherin overexpression mobilizes the protective effects of mesenchymal stromal cells against ischemic heart injury through a β-catenin-dependent manner. Circ. Res..

[39] Dews M (2006). Augmentation of tumor angiogenesis by a Myc-activated microRNA cluster. Nat. Genet..

[40] Jin R, Sun W, Bai Y, Huang LJ, Qu FJ (2019). Inhibitory effect of rapamycin on proliferation of human umbilical arterial smooth muscle cells. Immunopharmacol. Immunotoxicol..

[41] Yasui M (2006). Antisense to cyclin D1 inhibits vascular endothelial growth factor-stimulated growth of vascular endothelial cells: Implication of tumor vascularization. Clin. Cancer Res..

[42] Wagner N, Wagner KD (2020). PPARs and angiogenesis-implications in pathology. Int. J. Mol. Sci..

[43] Holm A, Heumann T, Augustin HG (2018). Microvascular mural cell organotypic heterogeneity and functional plasticity. Trends Cell Biol..

[44] Zauhar R (2022). As in real estate, location matters: Cellular expression of complement varies between macular and peripheral regions of the retina and supporting tissues. Front. Immunol..

[45] Lee J-W (2021). KAI1(CD82) is a key molecule to control angiogenesis and switch angiogenic milieu to quiescent state. J. Hematol. Oncol..

[46] Giddabasappa A (2016). Axitinib inhibits retinal and choroidal neovascularization in in vitro and in vivo models. Exp. Eye Res..

[47] Strittmatter K, Pomeroy H, Marneros AG (2016). Targeting platelet-derived growth factor receptor β+ scaffold formation inhibits choroidal neovascularization. Am. J. Pathol..

[48] Gianni-Barrera R (2018). PDGF-BB regulates splitting angiogenesis in skeletal muscle by limiting VEGF-induced endothelial proliferation. Angiogenesis.

[49] Yang XM (2009). Role of PI3K/Akt and MEK/ERK in mediating hypoxia-induced expression of HIF-1alpha and VEGF in laser-induced rat choroidal neovascularization. Invest. Ophthalmol. Vis. Sci..

