# Supplementary material for: A novel retinoic acid drug, EYE-502, inhibits choroidal neovascularization by targeting endothelial cells and pericytes
Source: Sci Rep. 2023 Jun 27;13:10439. doi: 10.1038/s41598-023-37619-7 (PMC10300120; doi:10.1038/s41598-023-37619-7)

Supplementary information

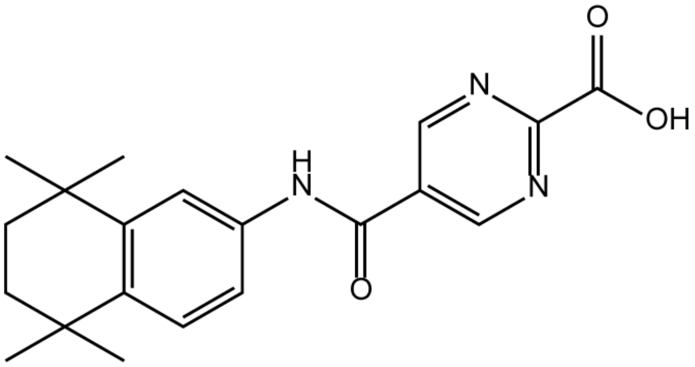

Figure S1. The structure of EYE-502

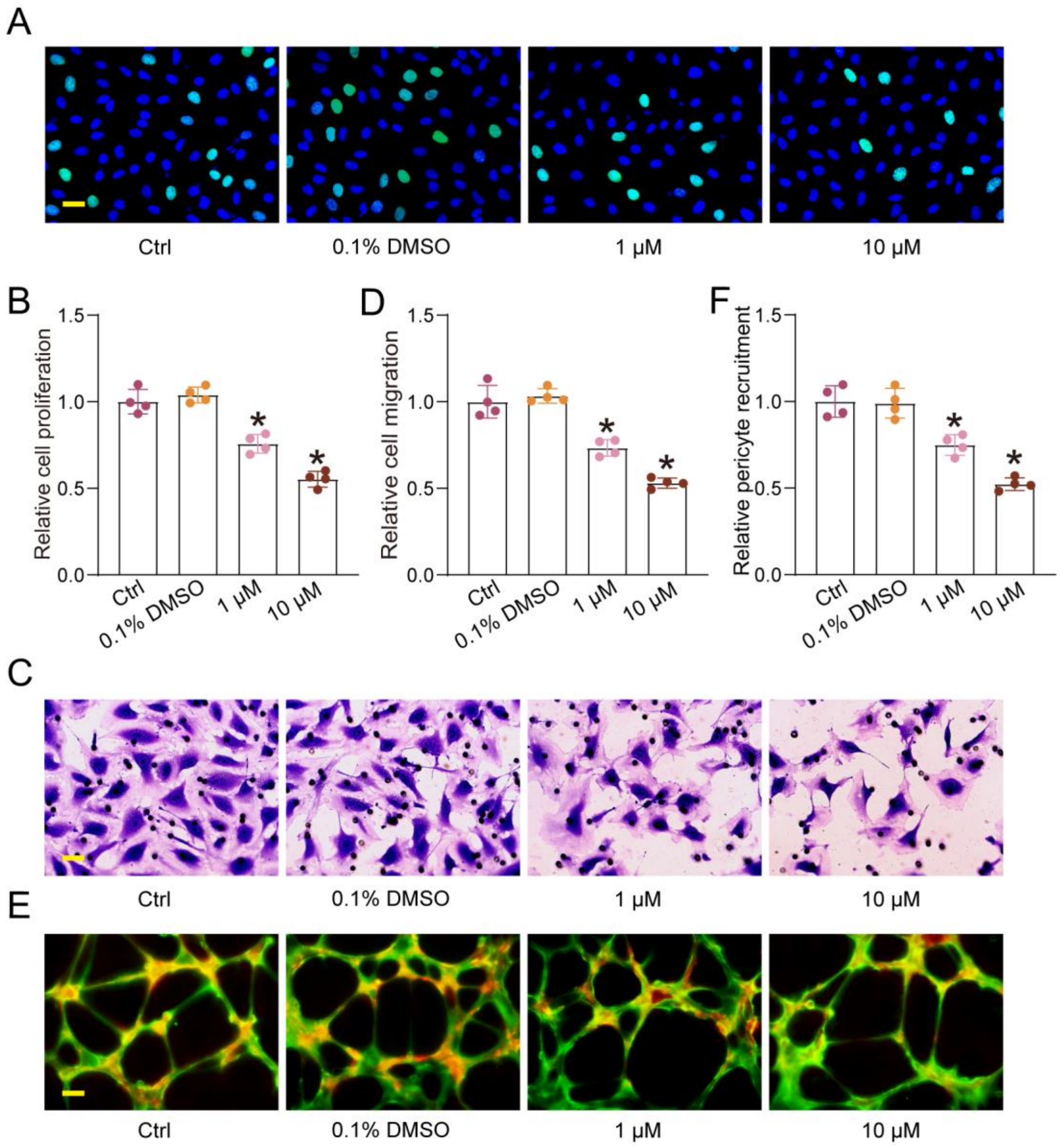

Figure S2. EYE-502 regulates choroidal pericyte function *in vitro*.

Primary choroidal pericytes were pretreated with PDGF-B (25 ng/ml), then exposed to EYE-502 (1 μM and 10 μM) or aflibercept for 24 h. The group pretreated with PDGF-B only was taken as the Ctrl group. (A - B) Cell proliferation was detected by EdU staining. Scale bar, 20 μm (n = 4). (C - D) Transwell assays were conducted to determine cell migration abilities. Scale bar, 20 μm (n = 4). (E - F) Primary choroidal pericytes were co-cultured with CECs on the matrigel matrix for 12 h and then stained with NG2 and CD31 to detect the recruitment of pericytes toward CECs. Scale bar, 50 μm (n = 4). * $P < 0.05$ versus Ctrl group. The significant difference was determined by one-way ANOVA followed by Bonferroni test.

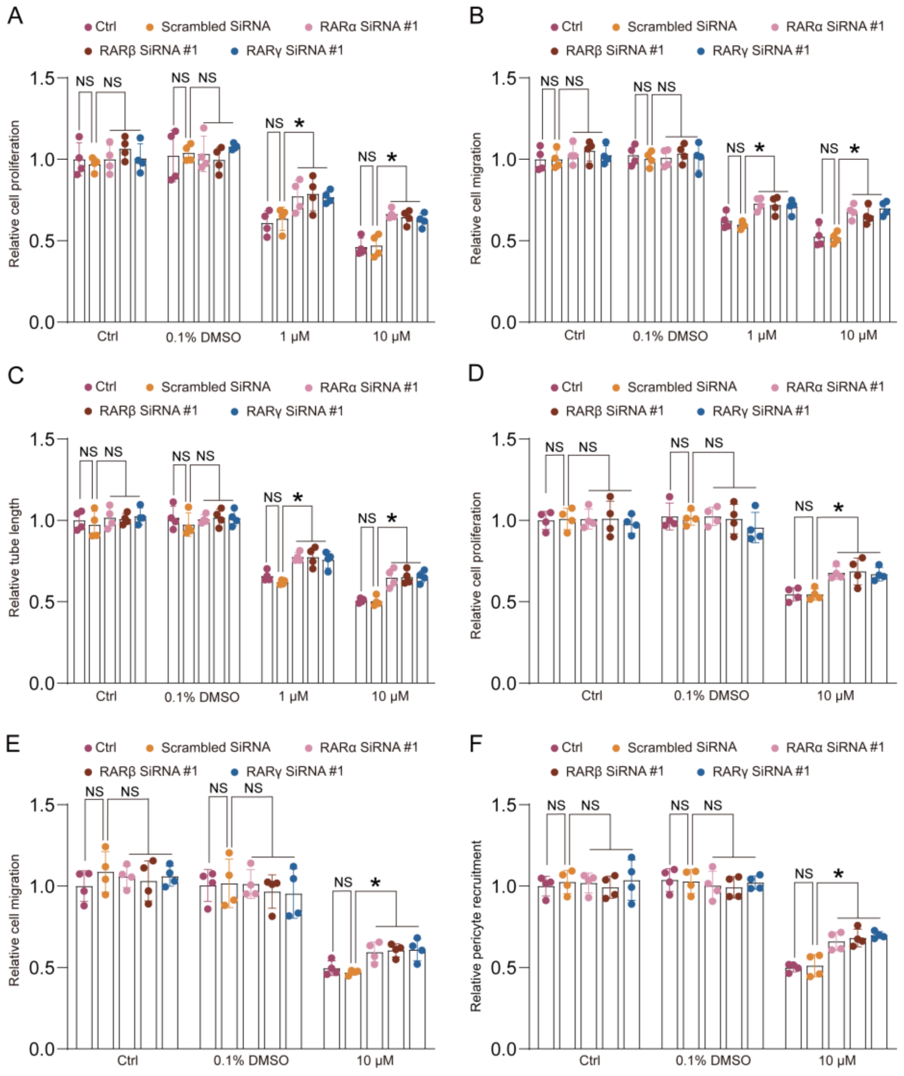

Figure S3. RARs are the primary cellular targets for EYE-502

(A - C) CECs were transfected with different siRNAs (scrambled siRNA, RARα siRNA, RARβ siRNA, or RARγ siRNA) or left untreated (Ctrl) for 24 h. After transfection, these cells were pretreated with VEGF (10 ng/ml) for 24 h, then exposed to 0.1% DMSO, EYE-502 (1 μM and 10 μM), or left untreated (Ctrl) for 24 h. EdU incorporation assays were conducted to detect cell proliferation (A, n = 4). Transwell assays were conducted to detect cell migration (B, n = 4). Matrigel-based tube formation assays were used to evaluate the formation ability of new vessels (C, n = 4). (D - F) Primary choroidal pericytes were transfected with siRNAs or left untreated (Ctrl) for 24 h. After transfection, these cells were pretreated with PDGF-B (25 ng/ml) for 24 h, then exposed to 0.1% DMSO, EYE-502 (1 μM and 10 μM), or left untreated (Ctrl) for 24 h. Cell proliferation was detected by EdU assay (D, n = 4). Transwell assays were conducted to determine cell migration (E, n = 4). Primary choroidal pericytes were co-cultured with CECs for 12 h and then stained with NG2 (pericytes) and CD31 (CECs) to detect the recruitment of pericytes toward CECs (F, n = 4). *$P < 0.05$ between the marked group; *NS* indicated no significant difference; One-way ANOVA followed by Bonferroni test.

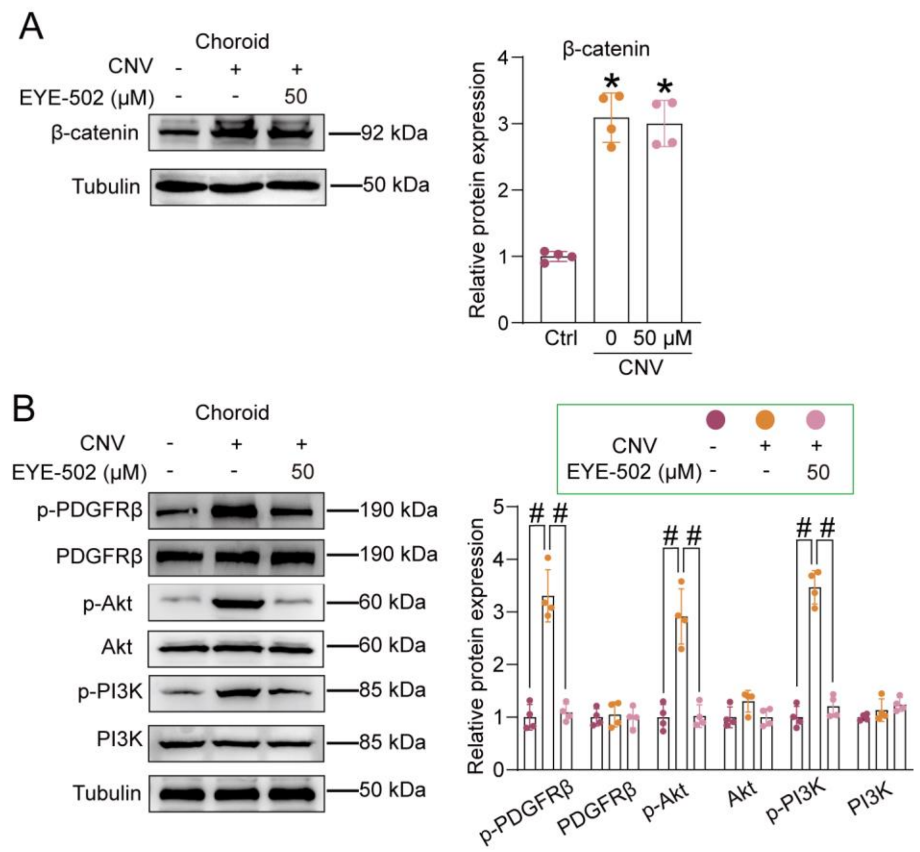

Figure S4. EYE-502 exerts its anti-angiogenic effects via Wnt signaling and PDGF signaling

(A, B) After CNV induction, C57BL/6 mice received intravitreal injections of PBS, 0.1% DMSO, or EYE-502 (50 μM). On day 14 after laser photocoagulation, the proteins of RPE/choroid complex were extracted. Western blots were conducted to detect the levels of total β-catenin, p-PDGFRβ, PDGFRβ, p-PI3K, PI3K, p-Akt, and Akt (n = 4). * $P < 0.05$ versus Ctrl group; # $P < 0.05$ between the marked group; One-way ANOVA followed by Bonferroni test.

Original gels/blots for Figure S4A

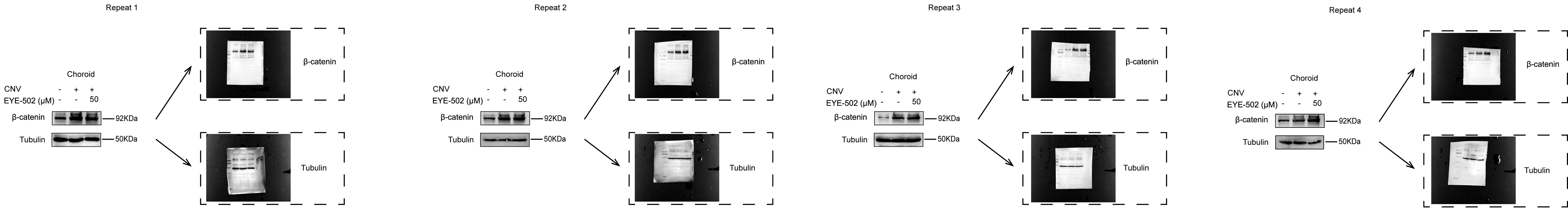

Original gels/blots for Figure S4B

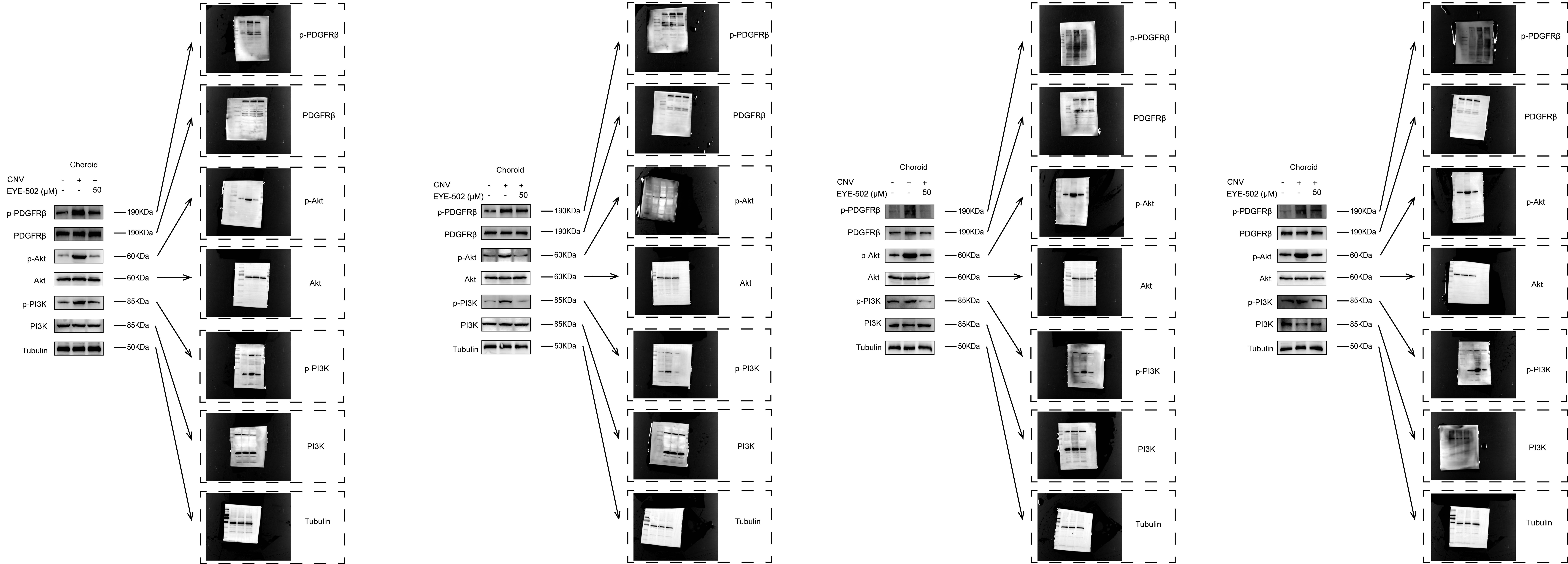

Original gels/blots for Figure 8A

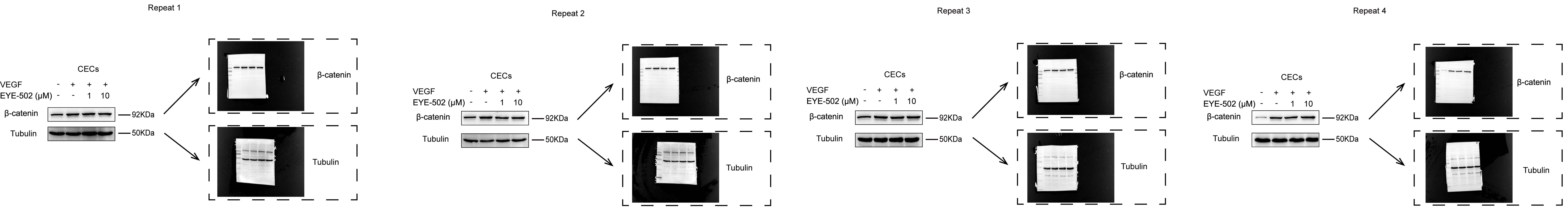

Original gels/blots for Figure 8D

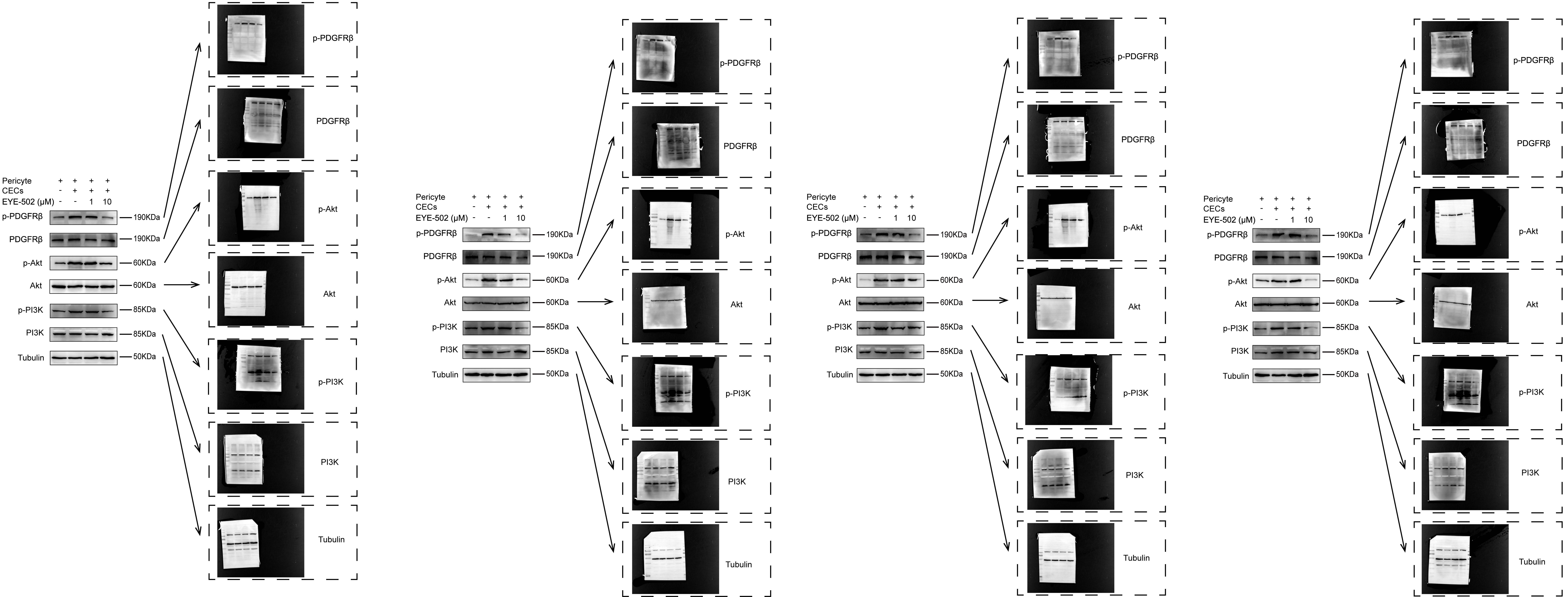

Supplement: Supplementary file 1 — Supplementary Information. [file 41598_2023_37619_MOESM1_ESM.pdf]
